# Assessment of lymph node area coverage with total marrow irradiation and implementation of total marrow and lymphoid irradiation using automated deep learning-based segmentation

**DOI:** 10.1371/journal.pone.0299448

**Published:** 2024-03-08

**Authors:** Hyeon Seok Choi, Hyun-Cheol Kang, Eui Kyu Chie, Kyung Hwan Shin, Ji Hyun Chang, Bum-Sup Jang

**Affiliations:** 1 Department of Radiation Oncology, Seoul National University Hospital, Seoul, South Korea; 2 Department of Radiation Oncology, Seoul National University College of Medicine, Seoul, South Korea; Chung-Ang University Gwangmyeong Hospital, REPUBLIC OF KOREA

## Abstract

**Background:**

Total marrow irradiation (TMI) and total marrow and lymphoid irradiation (TMLI) have the advantages. However, delineating target lesions according to TMI and TMLI plans is labor-intensive and time-consuming. In addition, although the delineation of target lesions between TMI and TMLI differs, the clinical distinction is not clear, and the lymph node (LN) area coverage during TMI remains uncertain. Accordingly, this study calculates the LN area coverage according to the TMI plan. Further, a deep learning-based model for delineating LN areas is trained and evaluated.

**Methods:**

Whole-body regional LN areas were manually contoured in patients treated according to a TMI plan. The dose coverage of the delineated LN areas in the TMI plan was estimated. To train the deep learning model for automatic segmentation, additional whole-body computed tomography data were obtained from other patients. The patients and data were divided into training/validation and test groups and models were developed using the “nnU-NET” framework. The trained models were evaluated using Dice similarity coefficient (DSC), precision, recall, and Hausdorff distance 95 (HD95). The time required to contour and trim predicted results manually using the deep learning model was measured and compared.

**Results:**

The dose coverage for LN areas by TMI plan had V100% (the percentage of volume receiving 100% of the prescribed dose), V95%, and V90% median values of 46.0%, 62.1%, and 73.5%, respectively. The lowest V100% values were identified in the inguinal (14.7%), external iliac (21.8%), and para-aortic (42.8%) LNs. The median values of DSC, precision, recall, and HD95 of the trained model were 0.79, 0.83, 0.76, and 2.63, respectively. The time for manual contouring and simply modified predicted contouring were statistically significantly different.

**Conclusions:**

The dose coverage in the inguinal, external iliac, and para-aortic LN areas was suboptimal when treatment is administered according to the TMI plan. This research demonstrates that the automatic delineation of LN areas using deep learning can facilitate the implementation of TMLI.

## Introduction

Total marrow irradiation (TMI) and total marrow lymphoid irradiation (TMLI) are both treatments for conditioning regimens prior to allogeneic and autologous hematopoietic stem cell transplantation for treating blood cancers (e.g., leukemia, lymphoma, and myeloma [1]). Total body irradiation (TBI) is a conventional treatment for the same purpose [2]; however, it can cause multiple toxicities despite limited prescribed radiation doses. Although it affords certain advantages over a chemotherapy monotherapy regimen [3], TBI usage has declined [4] due to its toxicity and the necessity for specialized facilities. However, advances in radiotherapy (RT) techniques have reduced the exposure of organs at risk (OAR) to radiation [5]. For example, in some clinical studies, TMLI has shown promising results, such as lower incidence of radiation-related toxicity and extramedullary relapse [6]. This demonstrates the potential for increasing prescription doses to target lesions [7].

Both TMI and TMLI can be used as pretreatments; however, a major difference is observed between them. The former only targets the bone marrow, whereas the latter targets both the bone marrow and major lymph nodes (LNs). Despite these different target lesions, the clinical variation between TMI and TMLI remains unknown; further research is required to compare these therapies. Moreover, planning and conducting clinical studies for TMI and TMLI is challenging due to their low prevalence and the lengthy follow-up required to demonstrate significant differences. Therefore, in such cases, it may be cautiously posited that clinical differences can be indirectly inferred through differences in dose distribution.

And if clinical differences exist, the delineation of the whole-body bone marrow, LN areas, and OARs is essential for TMLI treatment planning. However, this planning process is time-consuming. One study reports that approximately 12–16 h per patient are necessary [8]. Hence, the process is labor-intensive and poses a challenge for clinical adoption Recent advancements in RT have increasingly utilized deep learning technologies. These include applications in generating planning CT data from diagnostic MRIs [9], assisting in target contouring [10], creating treatment plans [11], and predicting radiotherapy outcomes [12]. A noteworthy aspect of this evolution is the significant time reduction in target contouring achieved through deep learning, as confirmed in a study [10]. This progress suggests a potential decrease in the time required for contouring in TMLI. The “nnU-NET” framework, prominent among deep learning models in these studies, stands out for its excellent performance and user-friendliness, making it accessible even to those without expert-level knowledge in computer programming [13].

In this study, dose coverage is estimated by delineating whole-body LN areas based on the computed tomography (CT) data of patients treated according to the TMI plan. Furthermore, a deep learning model is trained and evaluated for auto-contouring whole-body LN areas using a deep learning framework named “nnU-NET”.

## Materials and methods

### Patients and target structure delineation

In this study, the CT data from patients treated with TMI in 2017 and the CT data of patients who underwent whole-body CT scans from January 1, 2021 to December 31, 2021 at our institution were collected. The median age of the patients at the time of TMI was 40 years (interquartile range (IQR): 28–54 years), and 85% were male; 69% were diagnosed with leukemia. The median height, weight, and body mass index (BMI) of the patients who of the TMI patients were 170.8 cm (IQR: 164.8–175.8 cm), 69.1 kg (IQR: 63.5–80.3 kg), and 23.9 kg/cm^2^ (IQR: 23.3–25.7 kg/cm^2^), respectively. Regarding the prescribed doses for the 13 patients, one received 8 Gy in 4 fractions, another received 12 Gy in 4 fractions, and the remaining 11 patients received 10 Gy in 5 fractions.

To train the deep learning model, data were collected from 13 patients who underwent whole-body CT at our institution between January 1, 2021, and December 31, 2021. The median age of patients at the time of the CT scan was 64 years (IQR: 56–72 years, with 46% being male, and 69% undergoing whole-body CT for multiple myeloma diagnosis. Their median height, weight, and BMI were 162.25 cm (IQR: 150.75–164.5 cm), 61 kg (IQR: 53.5–68.5 kg), and 25.1 kg/cm^2^ (IQR: 23.4–25.7 kg/cm^2^), respectively. All collected CT data were non-contrast enhanced CT scans and the average pixel size was 1.113mm x 1.113mm, and the slice thickness was consistently 5mm.

Eighteen LN areas were manually delineated on each patient’s CT data by a radiation oncologist and then confirmed by two radiation oncologists. These LN areas included the cervical (Lt/Rt), axillary (Lt/Rt), and mediastinal LNs (Sup. /Inf.); para-aortic LN area; common iliac LN area (Lt/Rt); external iliac LN area (Lt/Rt); internal iliac LN area (Lt/Rt); obturator LN area (Lt/Rt); presacral LN area; and inguinal LN area (Lt/Rt). To delineate each LN area, delineation guidelines for head and neck, breast, lung, and cervical cancers were referenced [14–19].

### Dose estimation for LN areas treated by TMI

To calculate the dose received by each LN area using the TMI plan, information from patients who underwent TMI was used. Dose planning from previous TMI treatments was reloaded, and a manually delineated LN area with margins(5mm) for planning target volume was used in each plan to calculate the dose for each area. For each patient, Vx%, which is the volume of the organ receiving x% or more of the prescription dose, was calculated.

### Development of deep learning model

To train the deep learning model for medical image segmentation tasks, the “nnU-Net” framework is utilized [13]. This framework is characterized by its ability to function without extensive data pre-processing or parameter tuning. This is achieved through an “out-of-the-box” approach that uses a pretrained network and requires only minimal fine-tuning of target data. Hence, the framework is an ideal choice for users who lack expertise in image segmentation and deep learning. The “nnU-Net” framework has been extensively evaluated in a variety of imaging tasks and shown to achieve state-of-the-art performance especially in medical imaging tasks. The patients from whom CT data were collected were divided, with 16 individuals allocated to the training group, 4 to the validation group, and 6 to the test group. Training was performed by fivefold cross-validation using a 4:1 split of CT data from the training/validation group; the model with the best evaluation parameter value was selected. Training and testing were performed using a workstation equipped with an NVIDIA RTX A6000 with 48GB of graphics processing unit memory.

The models developed based on “nnU-NET” in this study are available in the Zenodo repository (doi.org/10.5281/zenodo.7839889).

### Model evaluation

**Fig 1:**
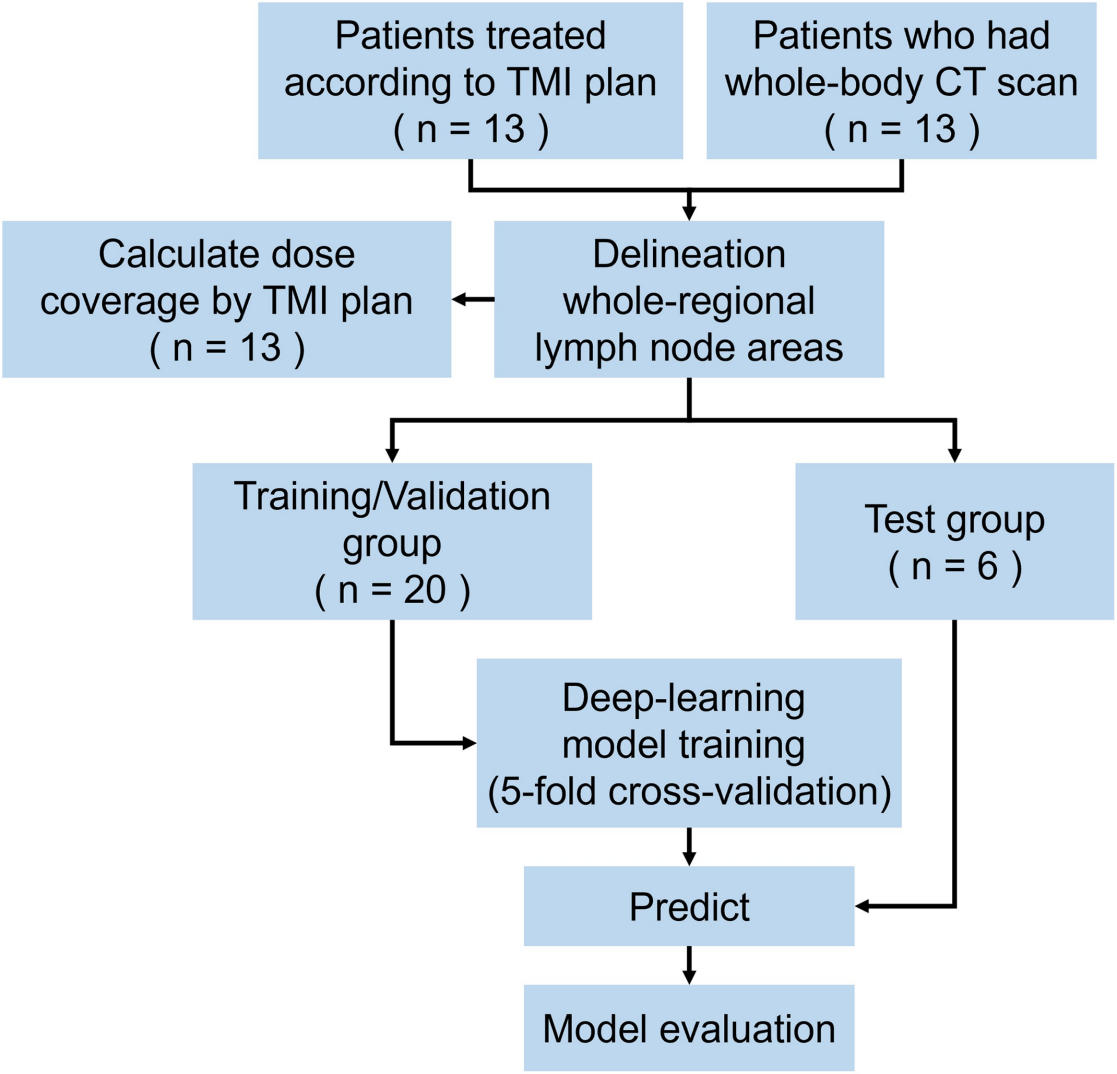
Study schema. The CT data of 26 patients were used. The patients were divided into 20 and 6 as training/validation and test groups, respectively. Abbreviations—TMI: total marrow irradiation; CT: computer tomography.

The trained models were evaluated using dice similarity coefficient (DSC), precision, recall, and Hausdorff distance 95 (HD95). The DSC is used to quantify the overlap between an observer and consensus contours; it is commonly employed as an index for image segmentation evaluation. The coefficient ranges from 0 (indicating no similarity) to 1 (indicating a perfect match). When the volume of ground truth is “G” and the volume of prediction by a trained deep learning model is “P,” the DSC, precision, and recall are calculated as 2×|*G*∩*P*|/(|*G*|+|*P*|), |*G*∩*P*|/|*G*|, and |*G*∩*P*|/|*P*|, respectively. To measure the distance between two sets, HD95 calculates the farthest point between the two. The HD95 approach is used because it is less sensitive to outliers. It measures the distance between two sets in which one set contains 95% or more of the points found in the other set. The evaluation metrics were calculated by aggregating values from LN areas, including both left/right and superior/inferior divisions. For the patients in the test group, the times necessary for manually delineating whole-body LN areas and accurately trimming the predicted LN areas are measured and then compared. This process is presented in Fig 1.

### Ethics statement

This study was approved by the Institutional Review Board of Seoul National University Hospital (IRB No. H-2204-050-1314). Personally identifiable information required for the CT data collection process was anonymized after collection. This study was approved by the IRB committee to waive consent because it is a retrospective medical record review study, obtaining participants’ consent is not practical at the time of the study, and the risk to participants of waiving consent is extremely low.

## Results

### Dose estimation for LN areas treated by TMI

**Fig 2:**
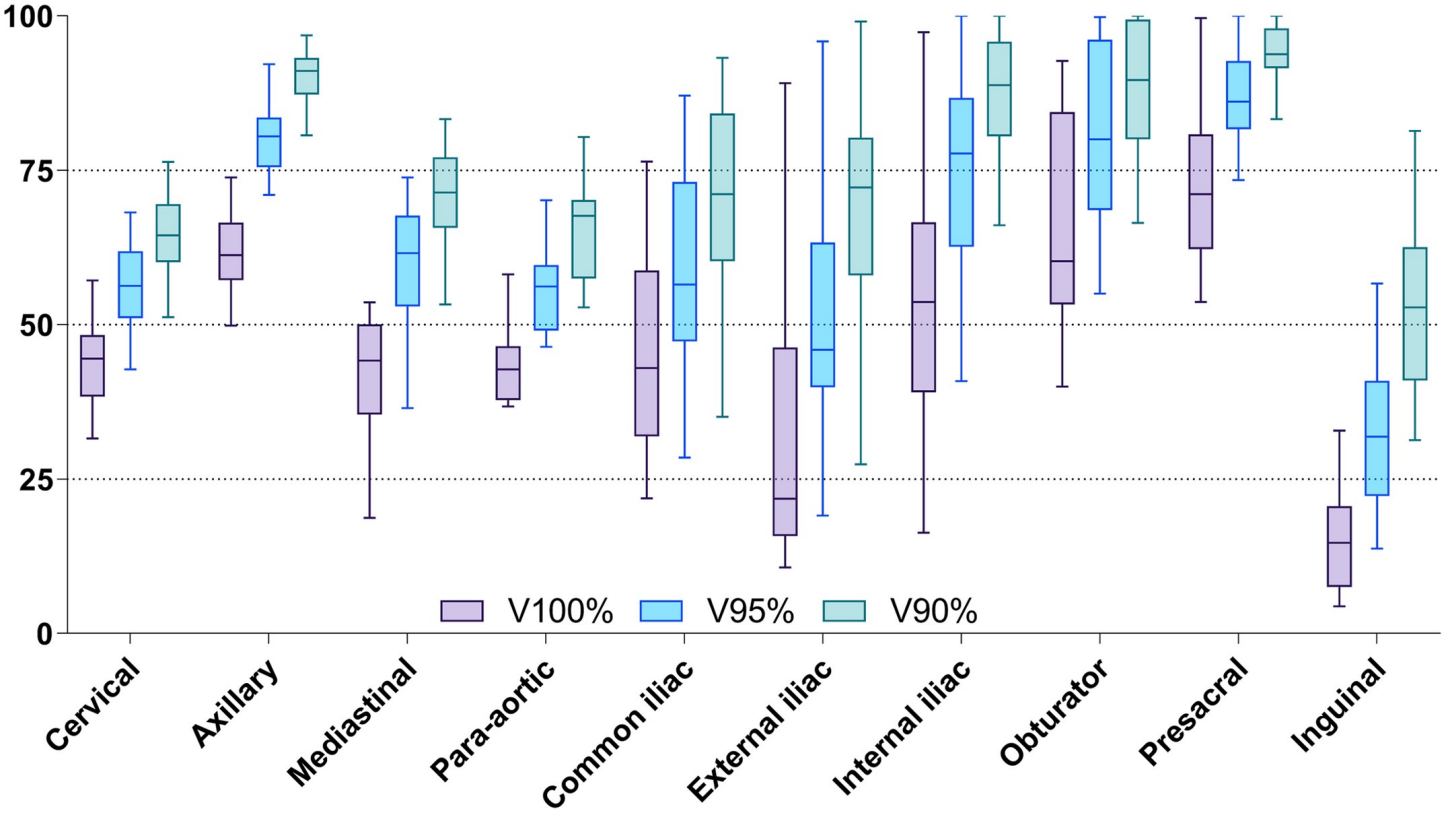
Dose coverage in each lymph node area. Box and whisker plot representation of the Vx% for each lymph node area. Abbreviations—Vx%: volume receiving x% or more of the prescription dose.

Table 1 and Fig 2 summarizes the dose distribution in the manually delineated LN using the TMI plan. The median values of V100%, V95%, and V90% of the entire manually contoured LN area were 46.5% (IQR: 33.0–58.7%), 62.4% (IQR: 49.5–78.3%), and 75.3% (IQR: 63.8–89.6%), respectively. The presacral, axilla, and obturator LN areas had relatively high V100% median values of 71.1% (IQR: 62.2–80.8%), 61.2% (IQR: 57.2–66.5%), and 60.3% (IQR: 53.3–84.4%), respectively. In contrast, the inguinal, external iliac, and para-aortic LN areas had relatively low V100% median values of 14.7% (IQR: 7.5–20.7%), 21.8% (IQR: 15.8–46.3%), and 42.8% (IQR: 37.8–46.5%), respectively. Similar trends were observed for V95% and V90%.

**Table 1:** Dose coverage in each lymph node area when delivering TMI plan.

LN area	V100% (%)	V95% (%)	V90% (%)
	(median (IQR))	(median (IQR))	(median (IQR))
Cervical	44.5 (38.3–48.4)	56.3 (51.1–61.9)	64.5 (60.1–69.6)
Axillary	61.2 (57.2–66.5)	80.5 (75.5–83.6)	91.1 (87.3–93.2)
Mediastinal	44.2 (35.5–50.1)	61.6 (53.0–67.7)	71.4 (65.7–77.1)
Para-aortic	42.8 (37.8–46.5)	56.2 (49.1–59.7)	67.6 (57.5–70.2)
Common iliac	43.0 (31.9–58.8)	56.5 (47.3–73.2)	71.1 (60.3–84.2)
External iliac	21.8 (15.8–46.3)	45.9 (39.9–63.3)	72.2 (58.0–80.3)
Internal iliac	53.7 (39.1–66.6)	77.7 (62.6–86.7)	88.8 (80.5–95.9)
Obturator	60.3 (53.3–84.4)	80 (68.6–96.2)	89.6 (80.0–99.4)
Presacral	71.1 (62.2–80.8)	86.1 (81.7–92.7)	93.8 (91.5–98.0)
Inguinal	14.7 (7.5–20.7)	31.9 (22.3–40.9)	52.8 (41.0–62.5)
Total	46.5 (33.0–58.7)	62.4 (49.5–78.3)	75.3 (63.8–89.6)

Abbreviations—TMI: total marrow irradiation; LN: lymph node; IQR: interquartile range; Vx%: volume receiving x% or more of the prescription dose.

### Development of deep learning model

**Fig 3:**
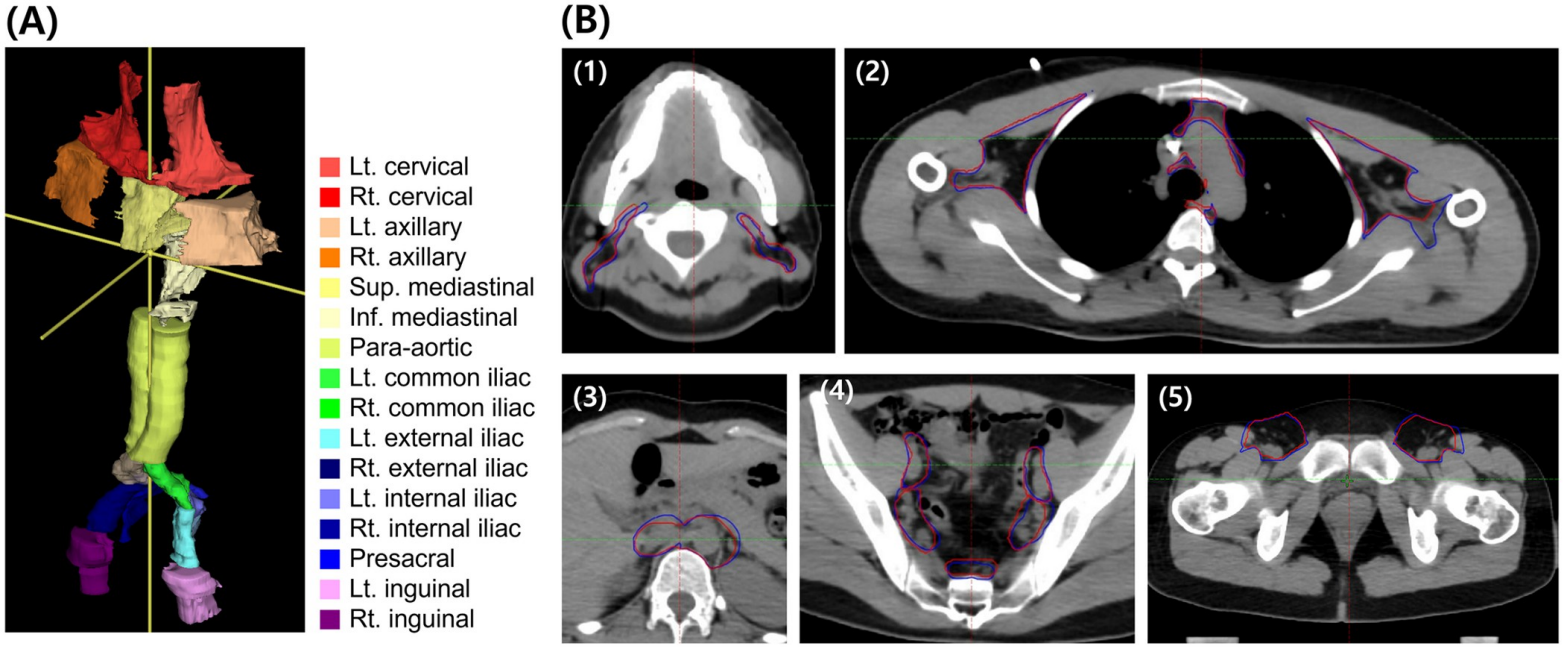
Delineated lymph node areas. (A) Manually delineated major lymph node areas of entire body of one patient with labels and (B) delineated lymph node area in one patient (blue lines denote manually contoured LNs, and red lines represent predictions obtained using deep learning): (1) cervical LN area; (2) axillary and mediastinal LN areas; (3) para-aortic LN area; and (4) iliac and presacral LN areas; (5) inguinal LN areas. Abbreviation—LN: Lymph node.

Out of a total of 26 patient datasets, 20 patients were assigned to the training/validation group. Within this group, the 20 patient datasets were divided in a 4:1 ratio to facilitate 5-fold cross-validation. The model that exhibited the highest performance during this cross-validation process was then selected. Following this selection, the best-performing model was further evaluated using the test group. Fig 3 shows an example of manually delineated LN areas versus the LN areas predicted by the deep learning model in a patient. The blue and red lines denote manually delineated areas and areas predicted using the deep learning model, respectively. Table 2 shows the mean volumes of manually delineated LN areas and predicted LN areas for patients in the test group by cc.

**Table 2:** Volumes of manually delineated and predicted lymph node areas.

Volume	Manually (cc)	Predicted (cc)
	(Mean±SD)	(Mean±SD)
Cervical	108.36±40.06	115.15±36.45
Axillary	131.23±36.65	138.66±36.63
Mediastinal	54.82±30.03	53.19±28.48
Para-aortic	318.45±67.85	259.33±90.92
Common iliac	37.56±8.23	29.63±11.95
External iliac	38.90±6.90	34.59±7.11
Internal iliac	30.85±4.56	30.40±9.03
Obturator	11.74±3.80	10.74±2.47
Presacral	23.02±8.81	19.09±11.22
Inguinal	108.08±28.62	98.76±32.37

Abbreviations—SD: Standard deviation

### Model evaluation

**Fig 4:**
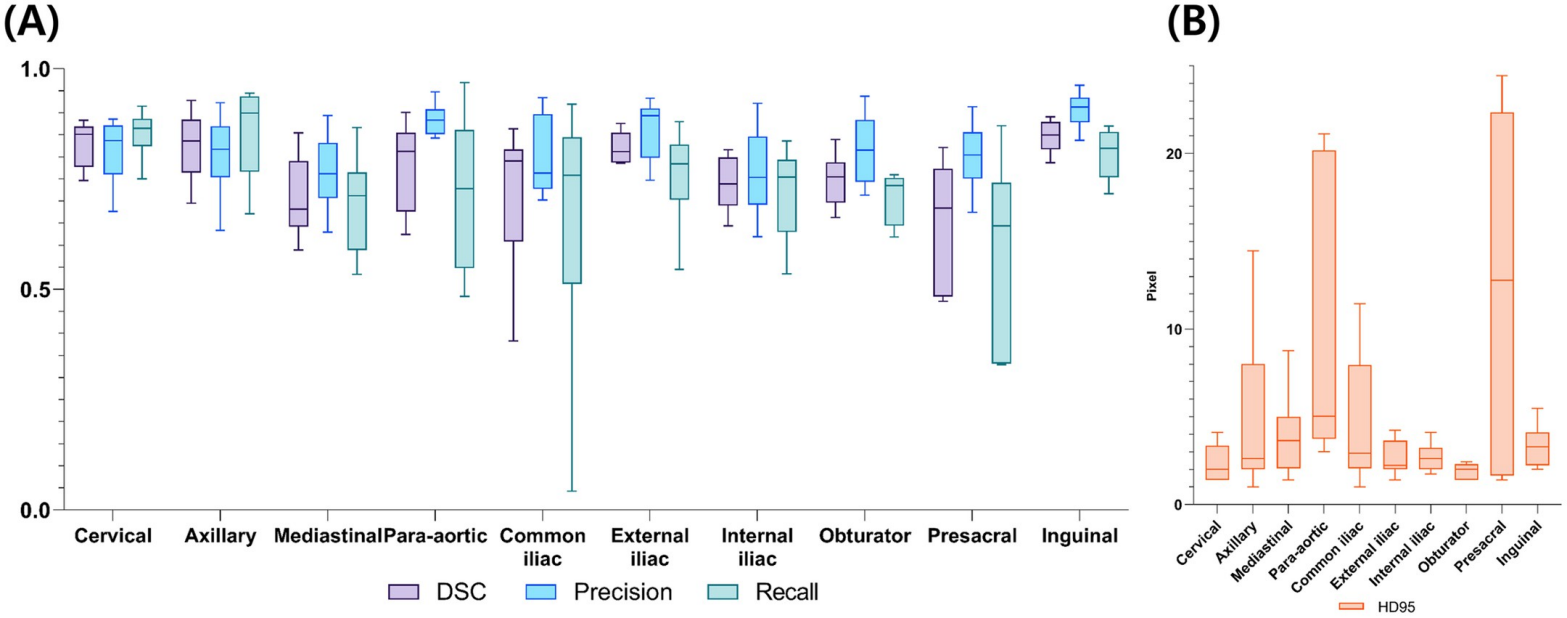
Evaluation results by lymph node area. (A) Dice similarity coefficient, precision, and recall of evaluation results; (B) Hausdorff 95 (HD95) values from evaluation results (unit: pixel). Abbreviations—DSC: Dice similarity coefficient; HD95: Hausdorff distance 95%.

Similarity scores between the manually delineated and predicted LN areas were estimated. As shown in Table 3 and Fig 4, the median values of DSC, precision, recall, and HD95 for the entire LN area are 0.79 (IQR: 0.70–0.84), 0.83 (IQR: 0.75–0.89), 0.76 (IQR: 0.68–0.85), and 2.63 pixels (IQR: 2.0–4.58 pixels). Among them, the cervical, inguinal, and axillary LN areas had high median DSC values of 0.85 (IQR: 0.78–0.87), 0.85 (IQR: 0.82–0.88), and 0.84 (IQR: 0.76–0.88), respectively. Conversely, the mediastinal, presacral and internal iliac LN areas had low median DSC values of 0.68 (IQR: 0.64–0.79), 0.68 (IQR: 0.48–0.77), 0.74 (IQR: 0.69–0.80), respectively. The precision was highest in the inguinal LN area, while the recall was highest in the axillary LN area. HD95 ranged from 2 and 3 pixels in most LN areas, with higher values indicating worse similarity. The presacral LN region and the para-aortic LN region had poor evaluation values (12.77 pixels (IQR: 1.65–22.32 pixels) and 5.04 pixels (IQR: 3.75–20.19 pixels), respectively).

**Table 3:** Evaluation results of predicted lymph node areas.

Parameter	DSC	Precision	Recall	HD95 (pixel)
	(Median (IQR))	(Median (IQR))	(Median (IQR))	(Median (IQR))
Cervical	0.85 (0.78–0.87)	0.84 (0.76–0.87)	0.86 (0.82–0.89)	2.00 (1.41–3.37)
Axillary	0.84 (0.76–0.88)	0.82 (0.75–0.87)	0.90 (0.77–0.94)	2.62 (2.00–8.02)
Mediastinal	0.68 (0.64–0.79)	0.76 (0.71–0.83)	0.71 (0.59–0.77)	3.64 (2.06–500)
Para-aortic	0.81 (0.68–0.86)	0.88 (0.85–0.91)	0.73 (0.55–0.86)	5.04 (3.75–20.19)
Common iliac	0.79 (0.61–0.82)	0.76 (0.73–0.90)	0.76 (0.51–0.84)	2.91 (2.06–7.95)
External iliac	0.81 (0.79–0.86)	0.89 (0.80–0.91)	0.78 (0.70–0.83)	2.24 (2.00–3.64)
Internal iliac	0.74 (0.69–0.80)	0.75 (0.69–0.85)	0.75 (0.63–0.79)	2.62 (2.00–3.24)
Obturator	0.75 (0.70–0.79)	0.82 (0.74–0.88)	0.74 (0.64–0.75)	2.00 (1.41–2.31)
Presacral	0.68 (0.48–0.77)	0.80 (0.75–0.86)	0.64 (0.33–0.74)	12.77 (1.65–22.32)
Inguinal	0.85 (0.82–0.88)	0.91 (0.88–0.94)	0.82 (0.75–0.86)	3.29 (2.24–4.12)
Total	0.79 (0.70–0.84)	0.83 (0.75–0.89)	0.76 (0.68–0.85)	2.63 (2.00–4.58)

Abbreviations—DSC: Dice similarity coefficient; HD95: Hausdorff distance 95%; IQR: interquartile range

Significant time differences between delineating the whole-body LN areas and trimming the predicted LN areas in the six test groups were observed (p < 0.001); the median times were 70′ 20″ (IQR: 64′ 54″–78′ 27″) and 30′ 16″ (IQR: 27′ 3″–32′ 37″), respectively.

## Discussion

This study aims to calculate the dose coverage of the LN area when treated according to the TMI plan, which has not been previously implemented. Results show that the inguinal, external iliac, and para-aortic LN areas have lower coverage than expected. This is probably due to the anatomical location of the LNs away from the bone. Targeting whole-body bones can be an appropriate pre-transplant conditioning regimen for hematopoietic stem cell transplantation given that most hematopoietic stem cells are present in the bone marrow. However, because malignant cells are also located in major LNs, additional targeting of an LN area may provide better clinical outcomes. Nevertheless, comparative studies have shown that TMI and TMLI have similar clinical outcomes as TBI [6]; however, but no prospective or retrospective studies comparing the clinical outcomes of TMI and TMLI have been conducted. Future studies are necessary to compare the TMI and TMLI plans in terms of the variable dose parameters of OARs and dose coverage.

The second objective of the current study is to develop a deep learning model for the automatic delineation of whole-body LN areas because manual delineation is a time-consuming and labor-intensive process when TMLI is delivered. A model is devised and subsequently trained and tested using the data from the 26 patients treated at the institution based on the “nnU-NET” deep learning framework. Although the amount of training data was small, the model performance and quality of the contours were acceptable for TMI planning. The predicted presacral and para-aortic LN areas are observed to have higher HD95 values than the other LN areas. This may be because these two LN areas have no laterality; consequently, the effect of training using few samples on these areas is less significant than that on LN areas with laterality. Nevertheless, the automatic delineation of whole-body LN areas using the developed model was demonstrated to be significantly more time-effective than manual delineation and had the potential to reduce the time required for total treatment planning.

Several papers on auto-contouring models using deep learning have been published, such as a model that auto-contours the target lesions of the head and neck, thorax, rectum, cervix, prostate, and heart structures [10,20–25]. In TMLI, target areas include bone and LN areas. For bone, due to the significant density difference with surrounding tissues, generally favorable results are observed [25]. Conversely, in LN areas, the density contrast with adjacent tissues is not as pronounced, leading to varied outcomes in different studies. Among these, Rhee et al. [22]. employed deep learning to automatically segment the Clinical Target Volume (CTV) in cervical cancer, achieving a mean DSC and HD95 of 0.81 and 2.09, respectively, for nodal CTV, and 0.76 and 2.00 for PAN CTV. Similarly, Cardenas et al. [21]. Developed an auto-segmentation model for LN CTV in head and neck cancer patients, yielding a mean DSC between 0.843 and 0.909. Our study demonstrated a median DSC of 0.79 and a median HD95 of 2.63, presenting a competitive performance in comparison with existing studies.

In contrast to these prevalent region-specific studies, whole-body auto-contouring models have received less attention in the current research landscape. Chen et al. [25]. Reported a model that can auto-contour whole-body organs. Most models utilize a limited range of CT data for each organ, or the entire body is divided into regions and trained separately to create each model. In contrast, a model based on whole-body CT data is developed in the current study; thus, a prediction for a body LN area can be immediately provided.

One study has shown that increasing the radiation dose in TBI results in better disease control [26]. Although disease control was improved, it was not linked to improved overall survival. This might be due to the toxicity of high doses of OARs. Thus, TMI and TMLI have been proposed as alternatives to reduce toxicity [5] despite the absence of published guidelines. Recently, Dei et al. reported the variability in TMLI target delineation [27]. The study found that inter-observer and intra-observer variabilities were reduced when guidelines for delineating each regional LN area were provided. Dei et al. provided documented guidelines for the current practice. Additionally, if a deep learning model can be used to provide patient-specific LN area delineation consistently, reduced variability in target delineation may be expected in TMLI planning.

One of the limitations of the current study is the small number of patients. In addition to the data of patients treated with TMI at our institution, whole-body CT data have been used as additional training data. This implies that the patient posture and image resolution between the RT and CT plans differ. Thus, further studies in the future are required for external validation using federated learning with other institutions. Another limitation of this study is the lack of international standards for TMLI target delineation. Even when the LNs are delineated according to the available contouring guidelines for several organs, concerns regarding the LN areas that may be targeted for TMLI persist. Additional fine-tuning of the model is necessary upon the availability of relevant guidelines.

## Conclusions

The dose coverage in the inguinal, external iliac, and para-aortic LN areas is suboptimal if a TMI treatment plan is implemented. The current research demonstrates that the automatic delineation of LN areas using deep learning can facilitate the implementation of the TMLI plan.

## Abbreviations

BMI: body mass index
CT: Computed tomography
DSC: Dice similarity coefficient
HD95: Hausdorff distance 95
IQR: interquartile range
LN: Lymph node
OAR: Organs at risk
RT: Radiotherapy
TBI: Total body irradiation
TMI—TMI: Total marrow irradiation
TMLI: Total marrow and lymphoid irradiation
